# An Integrated Approach Combining Chemical Analysis and an *In Vivo* Bioassay to Assess the Estrogenic Potency of a Municipal Solid Waste Landfill Leachate in Qingdao

**DOI:** 10.1371/journal.pone.0095597

**Published:** 2014-04-17

**Authors:** Yufeng Gong, Hua Tian, Lijia Wang, Suping Yu, Shaoguo Ru

**Affiliations:** 1 Marine Life Science College, Ocean University of China, Qingdao, China; 2 Qingdao Chengtou Xiaojianxi Leachate Treatment Corporation Limited, Qingdao, China; Massachusetts General Hospital, United States of America

## Abstract

Various adverse effects related to landfill leachate have made leachates an important issue in past decades, and it has been demonstrated that landfill leachate is an important source of environmental estrogens. In this study, we employed chemical analysis of some already evaluated estrogenic substances, in combination with a bioassay using several specific biomarkers (e.g., plasma vitellogenin and sex steroids, enzyme activity of gonad gamma-glutamyl transpeptidase, and gonadosomatic index) to evaluate the estrogenic activities in outlets from different stages of the leachate treatment process. The results indicated that 5 environmental estrogens (4-*t*-octylphenol, bisphenol A, di-ethyl phthalate, di-*n*-butyl phthalate, and diethylhexyl phthalate) were detected by a gas chromatography-mass spectrometry, and the concentrations in leachate samples were 6153 ng/L, 3642 ng/L, 2139 ng/L, 5900 ng/L, and 9422 ng/L, respectively. Leachate (1∶200 diluted) induced the synthesis of plasma vitellogenin and led to decreased enzyme activity of gonad gamma-glutamyl transpeptidase and gonadosomatic index in male goldfish (*Carassius auratus*) after a 28-day exposure, while increased circulating 17*β*-estradiol level was also observed in males exposed to treated effluent. Although the target EEs were partially removed with removal rates varying from 87.2% to 99.77% by the “membrane bioreactor+reverse osmosis+aeration zeolite biofilter” treatment process, the treated effluent is still estrogenic to fish. The method combined chemical techniques with the responses of test organisms allowing us to identify the group of estrogen-like chemicals so that we were able to evaluate the overall estrogenic effects of a complex mixture, avoiding false negative assessments.

## Introduction

Currently, sanitary landfill has become the most practiced method for managing waste disposal due to its economic advantages. In 2011, approximately 6.36 billion tons of municipal solid waste (MSW) was deposited in landfills in China [1]. Landfill leachate is generated by the infiltration and percolation of rainfall, groundwater, or runoff water into and through the waste layers [2] and is a complex mixture of inorganic and organic compounds. It has already been established as a hazard to the environment [3]. Taking the risk of leachate leakage into account, leachate-induced biotoxicity has become of concern to societies throughout the world [4]–[6]. The proposed endocrine disrupting effects, or more specifically estrogenic effects, caused by landfill leachate were reported in Sweden [7]–[9], and landfill leachate has been considered an important source of environmental estrogens (EEs) in the aquatic habitat [10].

Various examinations regarding EEs in leachate are being conducted, and to our knowledge, the measuring methods used can be mainly classified into two categories: chemical analysis and biological based assays. Chemical analysis is a rapid and sensitive screen to measure a large number of compounds. A variety of chemical analysis methods, such as gas chromatography-mass spectrometry (GC-MS) [11], [12], gas chromatography-flame ionization detector (GC-FID) [13], and high performance liquid chromatography (HPLC) [14], have been developed to identify estrogenic substances that potentially interfere with the reproductive system. Based on several combinations of instruments and detectors, the analytical results could provide excellent quantitative information of target compounds. However, it is limited in describing the overall estrogenic effects engendered by complex environmental samples containing multiple EEs because of the existence of additive, synergistic, or even antagonistic influences. Moreover, the pretreatment of chemical analysis may lead to the loss of some unknown EEs. These limitations can be overcome by utilizing biological responses of tested organisms, cellular, or even non-cellular materials in a biotest. Biological assays are typically classified as either *in vitro* or *in vivo* bioassays. Currently, several *in vitro* bioassays have been established and conducted for analyzing EEs, such as a simplified yeast two-hybrid assay [15], an E-screen test and enzyme-linked receptor assay [16], and a reporter-gene-based assay with a human breast-cancer-derived cell line [10]. There were also reports using chemical analysis and *in vitro* bioassays together to investigate the xenoestrogens in leachate [17]–[19], while *in vivo* bioassays have been rarely used in the examination of the biotoxicology of landfill leachate. Compared to the *in vivo* test, the *in vitro* bioassay introduced in previous studies has obvious limitations because it does not take into account biological accumulation, biotransformation, or disruptions at other target sites in the reproductive axis, which could potentially result in a false negative evaluation or disappreciation of the EE concentrations [20], [21]. For example, monocrotophos pesticide showed no estrogenic activity in the *in vitro* MCF-7 cell proliferation assay [22]; however, this pesticide’s *in vivo* estrogenic potency was confirmed by a goldfish vitellogenin (Vtg) induction experiment in our previous study [23].

For the *in vivo* bioassay, the use of multiple biomarkers at different biological levels can be an effective tool in providing comprehensive and available information on EEs. As a female-specific protein, Vtg is a recognized indicator of exposure to estrogen-like substances [24]–[26]. However, Kime et al. [27] gave a suggestion that the result of evaluation using Vtg as an exclusive biomarker should be treated with caution, and some other specific indicators should be included. The concentrations of plasma sex steroids could help to reveal Vtg induction mechanism(s) [25], [28], and enzyme activity of gonad gamma-glutamyl transpeptidase (*γ*-GTP) and gonadosomatic index (GSI) are always associated with the adverse effects on reproductive health of organisms, making them excellent complementary biomarkers for the indication of the presence of EEs [29], [30].

In the past few years, the estrogenic contamination in certain lake, river, effluents of municipal wastewater treatment plant, and sediment has been evaluated by using chemical analysis combined with *in vivo* bioassays [31]–[35]. For example, Ribecco et al. [35] suggested the presence of estrogen-like substances in the sediment by revealing an increase in the expression of genes encoding estrogen receptor in *Solea solea*, though the levels of the primary pollutants present in the sediment did not exceed permissible Italian levels. In the present study, a valid method combining GC-MS and an *in vivo* bioassay was established to assess estrogenic activity in a real case study: a MSW landfill site in Qingdao. Frequently reported potential estrogenic compounds (natural and synthetic steroid estrogens, alkylphenols, phthalic acid esters, and BPA) were measured in outlets from different stages of the leachate treatment process, and then the removal ratios were calculated. Plasma Vtg and sex steroids, testis *γ*-GTP enzyme activity, and GSI were selected as specific bio-indicators to investigate the estrogenic effects in a semi-static exposure experiment.

## Materials and Methods

### Selection of the MSW Landfill Site

**Figure 1:**
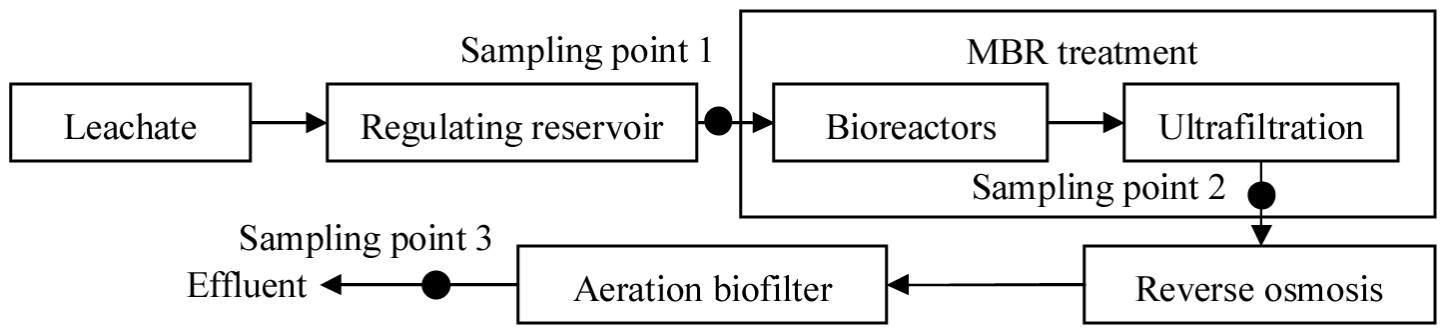
Schematic drawing of the treatment process for the leachate with sampling points (dots).

A MSW landfill site with a leachate treatment process in Qingdao was selected as a study model. It has been in operation since 2002 and mainly treats municipal solid waste and some bottom ash from MSW incinerators. The leachate treatment process used is an advanced membrane technology comprised of a membrane bioreactor treatment (MBR) with 4 bioreactors and an external ultrafiltration unit for sludge retention, a disctube reverse osmosis (DTRO) treatment, and a final aeration zeolite biofilter (see Figure 1). The leachate treatment facility is designed to collect and treat 900 m^3^ of leachate per day and the treated effluent discharged into the nearby river meets Grade one A standard of the *Discharge Standard of Pollutants for Municipal Wastewater Treatment Plant* (GB18918-2002, China). The technical data of the leachate and the treated effluent samples employed in the experiment are shown in Table 1.

**Table 1:** Technical data of leachate and treated effluent.

Technical data	Leachate	Treated effluent
COD_Cr_ (mg/L)	15000	≤20
BOD_5_ (mg/L)	5000	≤10
Ammonia nitrogen (mg/L)	2900	≤1
TN (mg/L)	3100	≤8
TP (mg/L)	15	≤0.5
TSS (mg/L)	2000	undetected

COD_Cr_: chemical oxygen demand; BOD_5_: five-day's biochemical oxygen demand; TN: total nitrogen; TP: total phosphorus; TSS: total suspended solids.

### Chemical Analysis

In September 2011, composite aqueous samples were collected in solvent-clean brown glass bottles from storage tanks at different sampling points as shown in Figure 1 for the detection of target EEs, including natural and synthetic steroid estrogens [estrone (E_1_), 17*β*-estradiol (E_2_), estriol (E_3_), and 17*α*-ethynilestradiol (EE_2_)], bisphenol A (BPA), alkylphenols [nonylphenol (NP), 4-*n*-octylphenol (4-*n*-OP), and 4-*t*-octylphenol (4-*t*-OP)], and phthalic acid esters [di-ethyl phthalate (DEP), diethylhexyl phthalate (DEHP), di-*n*-butyl phthalate (D_n_BP), and di-*n*-octylphthalate (DNOP)]. All samples were stored at 4°C, protected from light, and analyzed within 1 week.

Natural and synthetic steroids, alkylphenols, and BPA were extracted as previously described by Diniz et al. [33], with a few modifications. Briefly, samples (500 mL) were filtered through 0.45 µm glass fiber filters, mixed with 40 mL methanol and acidified with HCl to pH 3.0. Samples were then extracted through a 3 mL C18 solid phase extraction (SPE) cartridge which was previously conditioned with methanol and deionized water. EEs were eluted with 2×10 mL dichloromethane for steroids and BPA and with 2×10 mL methanol for alkylphenols. Elution fractions were evaporated to dryness under a gentle stream of nitrogen and reconstituted with 10% methanol to a final volume of 0.5 mL. The extraction of phthalic acid esters was adopted as follows: Samples (500 mL) were filtered through 0.45 µm glass fiber filters and extracted with 2×15 mL *n*-hexane for 10 min. The elution fractions were mixed and evaporated to a final volume of 0.5 mL.

The target compounds were analyzed using an Agilent 6890N/5973i GC-MS system (Agilent Technologies Inc., Palo Alto, USA). GC separations were carried out using an Agilent DB-17MS column (30 m×0.25 mm×0.25 µm). A sample volume of 1 µL was injected in splitless mode at an inlet temperature of 230°C. The mobile phase was He at a constant flow of 1 mL/min. For the detection of natural and synthetic steroids, alkylphenols, and BPA, the GC column temperature was programmed from 80°C (initial equilibrium time 2 min) to 230°C via a ramp of 15°C/min, 230°C–250°C via a ramp of 15°C/min, 250°C–280°C via a ramp of 5°C/min, and finally maintained at 280°C for 5 min. For the detection of phthalic acid esters, the GC column temperature was programmed from 60°C (initial equilibrium time 1 min) to 220°C via a ramp of 20°C/min, 220°C–280°C via a ramp of 5°C/min, and maintained at 280°C for 7 min. Identification of compounds was achieved by comparing the mass spectra and retention times of the chromatographic peaks with those of standard samples. Calibration curves were constructed by analyzing aliquots of a stock solution of authentic standards and all the correlation coefficients (*r^2^*) were above 0.99. Table 2 shows the relevant parameters of the GC-MS. The removal ratios were calculated as following: the removal ratio = (1–concentration of a certain detected compound in treated effluent/the corresponding concentration in leachate)×100%.

**Table 2:** Relevant parameters of the GC-MS.

EEs	linearequation	*r* ^2^	Qualificationions (m/z)	Quantitationions (m/z)	Recovery	LODs (ng/L)
E_1_	Y = 10.6X−18.7	0.9972	342	257, 218, 327	ND	10
E_2_	Y = 7.65X−16.0	0.9961	285	416, 232, 401	82.3	5
E_3_	Y = 2.05X−10.6	0.9946	285	368, 232, 218	ND	5
EE_2_	Y = 6.04X+40.9	0.9984	345	311, 504, 386	ND	3
4-*t*-OP	Y = 96.5X−1060	0.9984	207	208, 278, 209	112	3
4-*n*-OP	Y = 37.4X−292	0.9944	179	278, 180, 263	85.3	40
NP	Y = 23.3X+308	0.9994	179	180, 372, 193	190	40
BPA	Y = 86.3X−751	0.9954	357	372, 207, 179	235	40
DEP	Y = 237X−241	1.0000	121	149, 205, 223	ND	40
D_n_BP	Y = 443X−1620	1.0000	149	167, 205, 223	ND	10
DEHP	Y = 202X−1820	1.0000	113	149, 167, 279	82.3	5
DNOP	Y = 159X−2090	0.9996	149	167, 261, 279	ND	5

LODs: limits of detection; ND: not detected.

### *In vivo* Biological Assays

The fish were handled according to the National Institute of Health guidelines for the handling and care of experimental animals. The animal utilization protocol was approved by the Institutional Animal Care and Use Committee of the Ocean University of China. All surgery was performed under MS-222 anesthesia, and all efforts were made to minimize suffering.

Goldfish (*Carassius auratus*) are widespread throughout the world, and are a member of one of the largest vertebrate families, the *Cyprinidae*. Goldfish are omnivorous, feeding on plants, small crustaceans, and other food, which can reflect the food chain accumulation effect. In addition, many studies have been carried out to develop goldfish which could be handled easily and affordably in the laboratory, as a model animal for the evaluation of the estrogenic activities of various environmental chemicals [23], [36]–[38]. Male goldfish (9.4±0.8 cm standard length; 27.1±4.2 g wet mass) were obtained from a local dealer in Qingdao, China, and were acclimated in the laboratory for 14 days. To estimate possible estrogenic activity on male fish, we simulated an accidental leachate release into groundwater resulting in a 1∶200 dilution of the leachate; it was previously determined that a leachate dilution greater than 1∶200 resulted in mortality (see Table S1 for data). Based on these data, male fish were exposed to 0.5% diluted leachate with dechlorinated tap water (sampling point 1), 0.5% diluted MBR outlet with dechlorinated tap water (sampling point 2), and 100% treated effluent (sampling point 3) for the *in vivo* bioassay. The semi-static toxicity test was conducted in 70 L aquaria containing 50 L solution water. Each group of fish (n = 24) were exposed in three aquaria (8 fish/tank). Additionally, 20 L exposure solutions were renewed daily and a male negative control group (dechlorinated tap water) was also set up. Male fish with intramuscular injection of E_2_ dissolved in ethanol and peanut oil (1∶1, v/v) at a concentration of 5 mg/mL was used as the positive control. The water temperature was 23±2°C and the dissolved oxygen was 7.0±0.1 mg/L. Fish were fed a non-estrogenic pelletized diet daily and no death was observed during exposure.

After a 28-day exposure in September 2011, male goldfish were anesthetized in 75 mg/L MS-222 (Sigma, St. Louis, MO, USA). Blood was taken from the caudal vein using chilled heparinized syringes and then goldfish were dissected. After centrifugation (3500×*g*, 10 min), plasma was frozen in liquid nitrogen for the detection of Vtg protein and sex steroids. Testis samples were removed, weighed, frozen in liquid nitrogen and stored at −80°C for enzyme activity analysis.

Vtg is usually measured by immunoassay and detected in western blot using specific antibodies. In this study, the plasma vitellogenin analysis was carried out by western blot as previously described by Pan & Tian et al. [39]. Discontinuous sodium dodecyl sulfate-polyacrylamide gel electrophoresis (SDS-PAGE) was performed using a resolving gel with 9% acrylamide and a stacking gel with 4% acrylamide. Rabbit anti-goldfish Vtg serum (prepared in previous studies) diluted 1∶600 in blocking buffers and goat anti-rabbit IgG horseradish peroxidase conjugate (Beijing Solarbio Science & Technology Co., Ltd., Beijing, China) diluted 1∶1600 in blocking buffers were used to detect the plasma Vtg protein.

Radioimmunoassay kits obtained from the Beijing North Institute of Biology Technology, China, were used to detect the plasma E_2_ and testosterone (T) levels. The detection of plasma steroids was performed according to the manufacturer’s instructions. The assay detection limits were 10 pg/mL for E_2_ and 0.1 ng/mL for T. The inter- and intra-assay coefficients of variation for E_2_ and T were controlled under 8% and 10%. The interaction ratio for E_2_ and T detection with other hormones is <0.03%.

*γ*-GTP testing kits (Nanjing Jiancheng Bioengineering Institute, Nanjing, China) were used for the *γ*-GTP enzyme activity test. Gonads were homogenized in 9 volumes (w/v) of homogenate buffer on ice. The supernatant was prepared using centrifugation at 4000×*g* for 10 min at 4°C. Analyses of *γ*-GTP enzyme activity were performed on ice according to the testing kit instructions, and protein contents were measured according to Bradford’s method using bovine serum albumin (BSA) as the standard [40]. One unit (U) was defined as 0.01 OD produced by 1 mg protein in 1 h at 37°C. The assay detection limit was 0.08 U·mg/prot.

### Statistics

The SPSS (version 16.0; USA) was employed to process all data. The data are expressed as the mean ± standard deviation (SD). Multiple comparisons were performed using one-way ANOVA analysis after checking for normality and equal variance. All differences were considered significant at *p*<0.05.

## Results

### Occurrence of EEs

The occurrence of EEs in outlets from different stages of the treatment process during this study is summarized in Table 3. 5 environmental estrogens (4-*t*-OP, BPA, DEP, D_n_BP, and DEHP) were detected whereas natural and synthetic steroid estrogens, NP, and DNOP were under detection limits. The highest concentrations were found in leachate and reached 6153 ng/L, 3642 ng/L, 2139 ng/L, 5900 ng/L, and 9422 ng/L, respectively. The removal ratios of 4-*t*-OP, BPA, DEP, D_n_BP, and DEHP were 99.7%, 99.04%, 99.69%, 87.2%, and 92.9%, respectively.

**Table 3:** Mean concentrations of target EEs measured by GC-MS and EEQs of different samples.

Samples	Selected EEs (ng/L)					EEQ (ng/L)
	4-*t*-OP	BPA	DEP	D_n_BP	DEHP	
Effluent	14.3	34.8	6.66	757	666	0.01
MBR outlet	13.41	5.88	2.87	198.5	530	0.008
Leachate	6153	3642	2139	5900	9422	3.78

The estrogen equivalent quantity (EEQ) was defined as the sum of the concentration for each individual compound after multiplying an estradiol equivalency factor derived from previous studies [17], [73]–[76]. E_1_, E_2_, E_3_, EE_2_, and DNOP were under detection limits.

### Induction of Plasma Vtg

**Figure 2:**
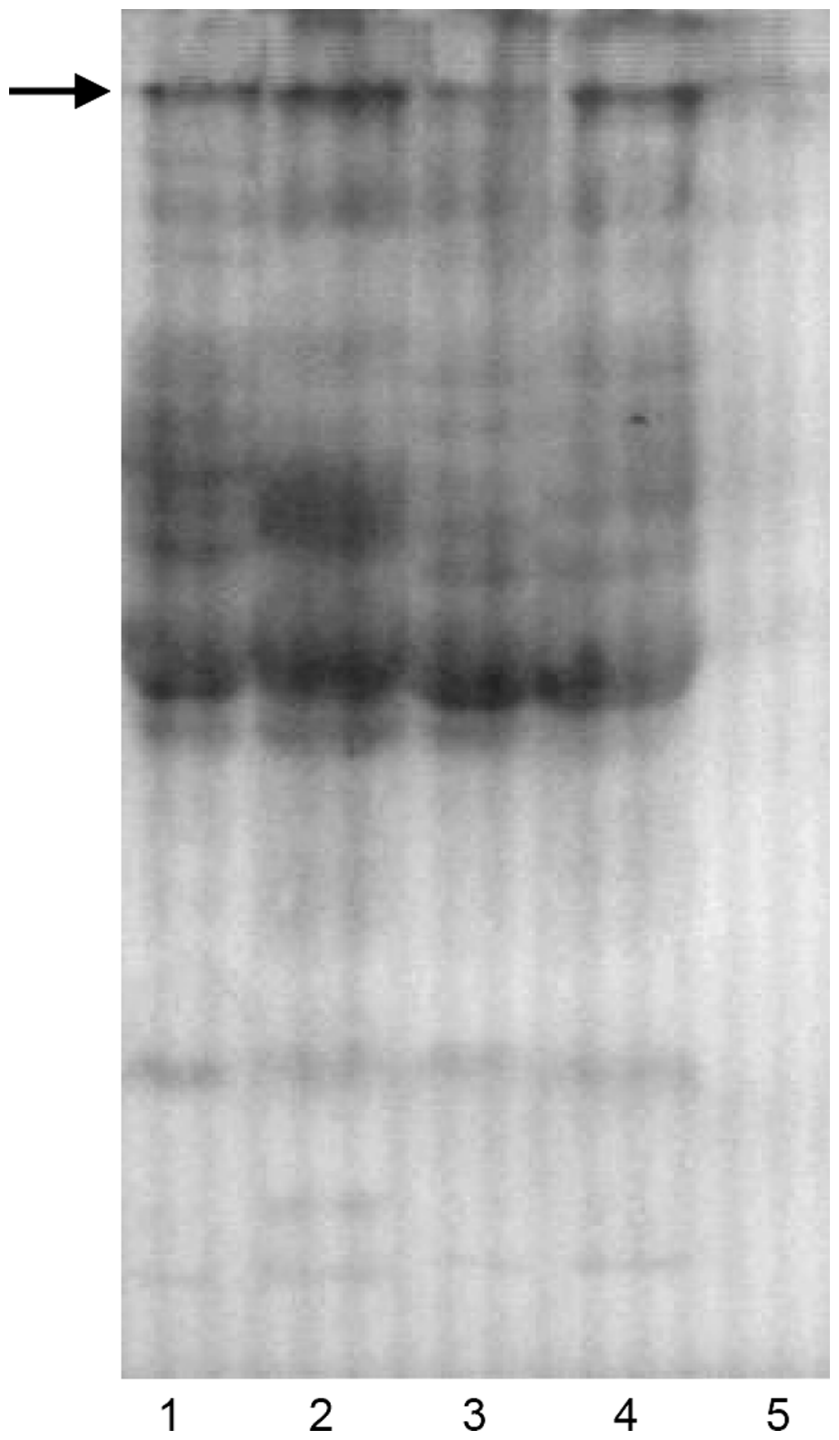
Western blot of plasma from treated and control male fish. Lane 1: plasma from E_2_ injected fish (20×); Lane 2: plasma from effluent exposed fish; Lane 3: plasma from 0.5% diluted MBR outlet exposed fish; Lane 4: plasma from 0.5% diluted leachate exposed fish; Lane 5: plasma from negative control male. Primary antibody: rabbit anti-goldfish Vtg serum (diluted 1∶600). Secondary antibody: goat anti-rabbit IgG horseradish peroxidase conjugate (diluted 1∶1600).

Plasma samples were analyzed for Vtg by western blot (Figure 2). The results revealed the presence of one Vtg protein subunit in the plasma of 0.5% leachate exposed fish and 100% treated effluent exposed fish, which was also observed in the E_2_ induced male but absent from the control male fish, indicating the induction of Vtg protein synthesis by 0.5% leachate and treated effluent exposure. In contrast, Vtg protein was absent from plasma of fish exposed to 0.5% MBR outlet.

### Effects of Sex Steroids Levels in Plasma

**Figure 3:**
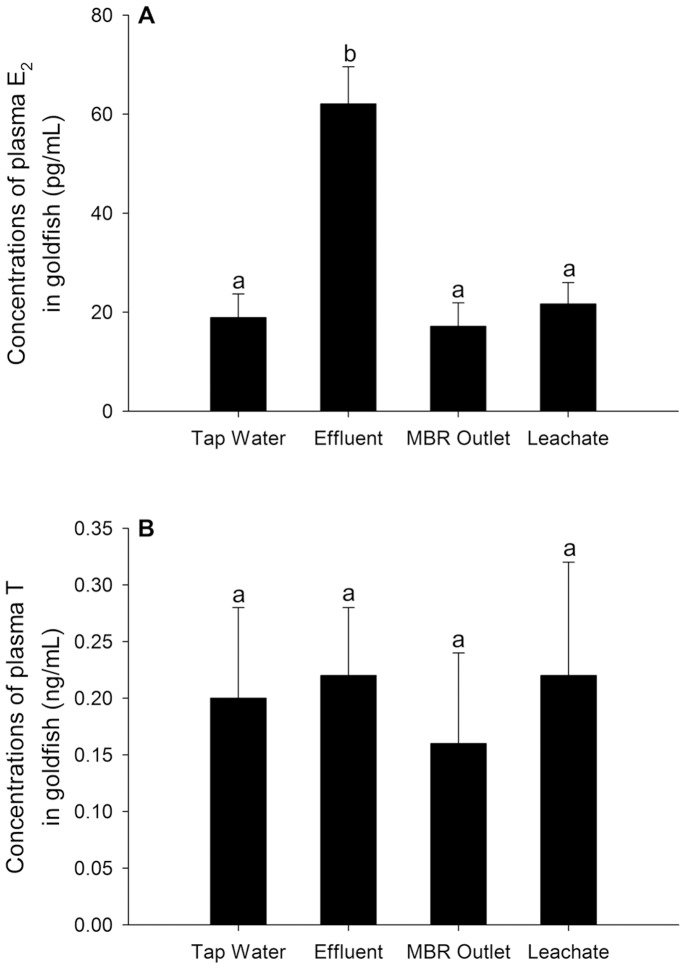
Quantification of plasma sex steroid levels in exposed male fish. Male goldfish were exposed to dechlorinated tap water, 100% treated effluent, 0.5% diluted MBR outlet, and 0.5% diluted landfill leachate (designated Tap Water, Effluent, MBR Outlet, and Leachate, respectively) for 28 days in a semi-static exposure system. Data are presented as the mean ± standard deviation (n = 24). Values with different letters denote statistically significant differences according to a one-way ANOVA analysis (*p*<0.05).

As shown in Figure 3A, the level of E_2_ in the plasma from treated effluent exposed fish (62.06±7.49 pg/mL) was significantly increased (*p*<0.05), while that of the other two groups was unchanged compared to the control male fish. Furthermore, the plasma T concentrations were not affected by any of the outlets from different stages of the treatment process (Figure 3B).

### Effects on Gonad γ-GTP Activity and GSI

**Figure 4:**
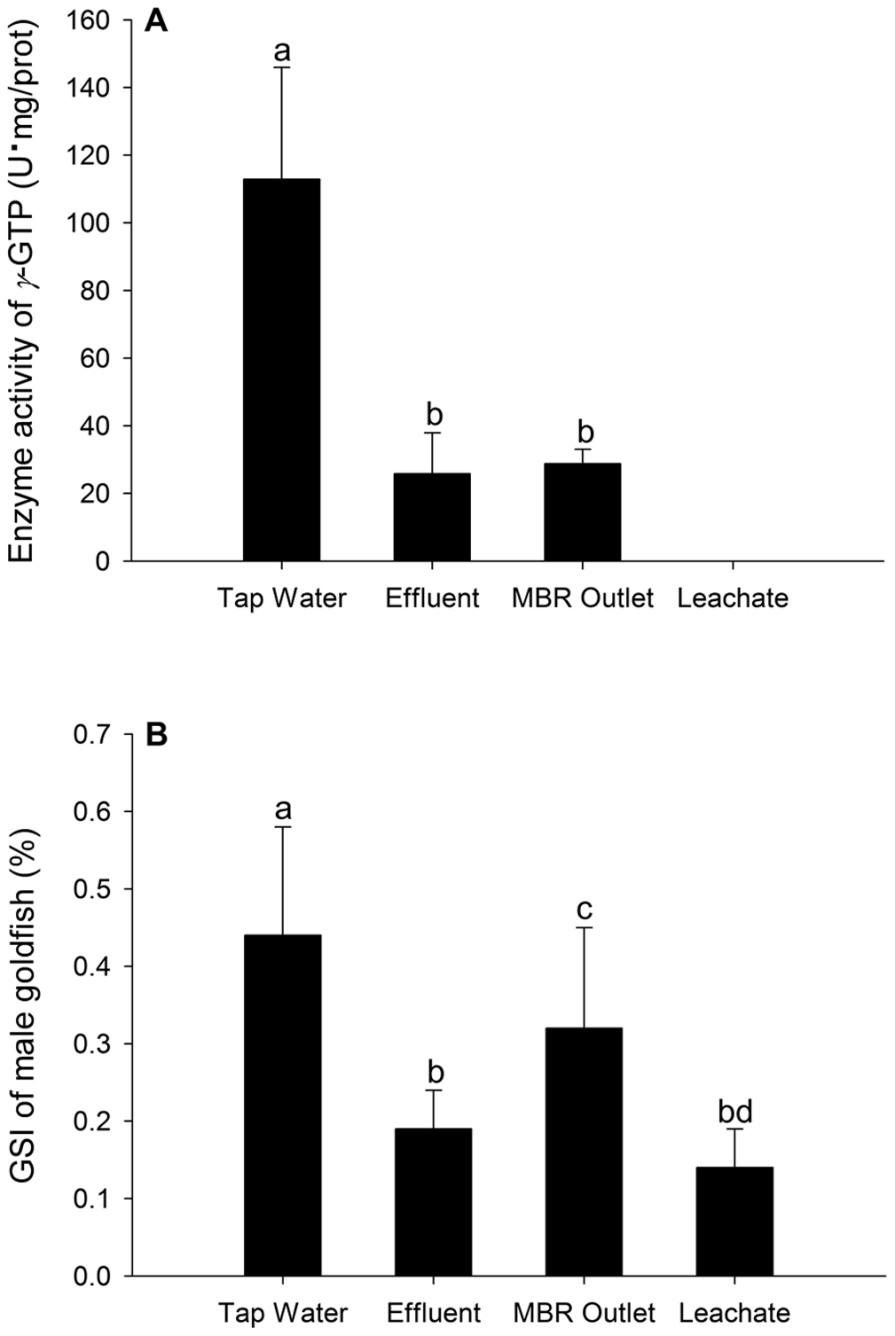
*γ*-GTP activity in gonad (A) and GSI (B) of exposed male goldfish. Male goldfish were exposed to dechlorinated tap water, 100% treated effluent, 0.5% diluted MBR outlet, and 0.5% diluted landfill leachate (designated Tap Water, Effluent, MBR Outlet, and Leachate, respectively) for 28 days in a semi-static exposure system. Data are presented as the mean ± standard deviation (n = 24). Values with different letters denote statistically significant differences according to a one-way ANOVA analysis (*p*<0.05).

Enzymatic activities of *γ*-GTP in the gonad were down-regulated after a 28-day exposure of outlets from different stages of the treatment process (Figure 4A), among which the *γ*-GTP activity in the male fish exposed to 0.5% leachate was close to the detection limit (0.08 U·mg/prot).

The gonadosomatic index (GSI) was calculated by: GSI = (total gonad weight/wet mass)×100%. As shown in Figure 4B, GSIs of male fish exposed to 0.5% leachate, outlet of MBR treatment, and treated effluent were significantly lower than control after a 28-day exposure.

## Discussion

Chemical analysis was utilized to identify the chemical components of leachate samples. Yasuhara et al. [41], [42] investigated approximately 190 types of chemical substances and more than 100 types of organic compounds including 1, 4-dioxane, phthalates, and bisphenol A in leachate samples in Japan. 41 sorts of phenols including low concentrations of 4-*t*-OP to high concentrations of BPA were detected in 38 municipal solid waste landfill sites [43]. Chemical analysis can serve as a useful tool to identify a group of EEs in leachate, by which the possible source of these compounds can be investigated. In this study, 5 identified EEs (4-*t*-OP, BPA, D_n_BP, DEP, and DEHP) were detected in leachate samples collected from selected landfill sites, and high detection rates have been frequently reported for them in previous studies [11], [12], [17]. 4-*t*-OP is the most representative final breakdown product of alkylphenol polyethoxylates (APEOs), which are widely used as wetting and emulsion agents in industrial and household applications [33]. BPA is employed during the production of polycarbonate and epoxy resins, which are important raw materials of commodity products [44]. PAEs are mainly applied as plasticizers in the production of dope, binder, and a variety of plastics [45]. It has been suggested that the occurrence of these detected estrogen-like chemicals may be a result of the degradation of household appliances, more importantly household plastics. In addition, the bottom ash from MSW incinerators could be another source [43].

Of more importance, chemical analysis is recommended as a prevailing methodological approach for the quantification of known or suspected EEs. In this study, measured concentrations of 5 types of estrogenic compounds were found to be present in concentration ranges reported in the literature. Teuten et al. [46] reported that the highest concentration range of BPA was 0.18–4300 µg/L in leachates from tropical Asian countries. Jonsson et al. [47] found the occurrences of DEHP (5–460 µg/L), D_n_BP (1–23 µg/L), and DEP (1–33 µg/L) in 17 European landfill leachates. However, the concentration of 4-*t*-OP found in this study was a little higher (0.03–3.4 µg/L by Teuten et al. [46]; 0.005–1.2 µg/L by Kurata et al. [43]; 0.04–0.31 µg/L by Asakura et al. [48]). The difference in 4-*t*-OP levels may be due to the composition of the landfill waste or the landfilling method.

Because of complex matrices of landfill leachate, the chemical analysis is limited in providing comprehensive knowledge of the overall estrogenic effects that might be engendered by leachate. This limitation can be overcome by the *in vivo* bioassay which utilized a series of specific biomarkers. In this study, a battery of bio-indicators including plasma Vtg and sex steroids, testis *γ*-GTP enzyme activity, and GSI were employed to evaluate the estrogenic activity at the molecular level, biochemical level, and tissue level.

Vtg is a female-specific phospholipoglycoprotein synthesized in the liver as nutrients for the developing embryo and is induced by endogenous estrogens [49], [50]. Male and juvenile fish normally produce minute amounts of Vtg due to the lack of endogenous estrogens. Therefore, an exposure to estrogenic compounds would lead to an abnormal induction of plasma Vtg, which makes it an ideal biomarker to test for exogenous estrogen exposure [24], [26], [51]. Also as mentioned in the preliminary draft guideline “The Fish Screening Assay for Endocrine Active Substances” within Organisation for Economic Co-operation and Development (OECD), Vtg is employed as a core biomarker endpoint for detecting endocrine active chemicals (unpublished). Currently, the Vtg induction assay has been widely used in assessing the estrogenic activity of effluent from wastewater treatment plants [52]–[54]. Our current study demonstrated that vitellogenin secretion was significantly induced in male goldfish after exposure to 0.5% diluted leachate and 100% treated effluent, suggesting significant estrogenic properties of these outlets *in vivo*. It is likely that estrogenic substances present in leachate and treated effluent were taken up by the fish and thereby stimulated the biosynthesis of Vtg. Considering the weak Vtg induction found in plasma of male fish exposed to 0.5% MBR outlet, the estrogenic activity was thought to be relatively low. Because the normal concentrations of sex steroids were not affected in 0.5% leachate for exposed male fish, it appears that various components of landfill leachate may bind to the estrogen receptor directly to induce Vtg synthesis. Evidence from *in vitro* assays has indicated that growth hormone and prolactin can also induce Vtg [55], [56].

Exposure to EEs is always associated with altered concentrations of plasma sex hormones, which play a decisive role in the regulation of sex differentiation, sexual characteristics, reproductive behavior, and reproductive capacity [8]. According to Lu et al. [32], male goldfish exposed to water from the Nanjing section of the Yangzi River had an elevated plasma E_2_ concentration. Similarly, plasma and testicular T levels in prepubertal male rats were decreased after subcutaneous administration of BPA, a recognized environmental estrogen, as described by Nakamura et al. [57]. In the present study, exposure of goldfish to treated effluent resulted in significantly increased circulating E_2_ levels compared to the negative control group, indicating a disturbance of sexual hormone homeostasis in male goldfish. The increased endogenous estradiol is one of the possible explanations of Vtg synthesis. In addition, this lower estrogenic potency of MBR outlet is further supported by the fact that the concentration of plasma E_2_ in 0.5% MBR exposed males was not affected. Endogenous E_2_ is mainly generated by the conversion of T [58] and the raised E_2_ level is always accompanied by a decreased T level. In the current study, exposure to 100% treated effluent for 28-days caused significant increases in plasma E_2_ levels but not in the T level, thus indicating the possible existence of androgenic activity. Studies in recent years have demonstrated that landfill leachate, municipal waste water, and industrial waste water may contain androgenic compounds [59]–[62]. For example, nine androgens and nine progestogens were detected in the influent and effluent of wastewater treatment plants in Beijing, China [63]. In addition, the biotransformation among androgens can be another reason for the unaffected T levels in plasma, as there are many types of androgens in male fish, such as androstenedione, 17*α*, 20*β*-algestone, and 11-ketotestosterone. Further detailed analysis is required to confirm this hypothesis.

The *γ*-GTP enzyme is mainly localized in the Sertoli cells of testis and the activity of the *γ*-GTP enzyme is a frequently used biomarker to indicate a lesion in the Sertoli cells and damage to the reproduction organs of male fish by xenobiotics at the biochemical level [30], [64]. Previous studies have suggested that exposure to 4-*t*-OP or NP may lead to structural damage of Sertoli cells and decreased enzyme activity of *γ*-GTP in eelpout (*Zoarces viviparus*), the effect of which is similar to E_2_ [65], [66]. Because the *γ*-GTP activity in testis of male fish exposed to treated effluent was decreased, it is reasonable to conclude that the Sertoli cells might be damaged due to the remarkable estrogenic potency. The enzyme activity of *γ*-GTP of male fish exposed to 0.5% leachate was close to the detection limit (0.08 U·mg/prot), which indicates the high biotoxicity of leachate due to the existence of hazardous substances.

GSI is a highly comprehensive index to assess the damage of reproductive function at the tissue level. Generally speaking, the exposure to environmental estrogens always results in a pronounced decline in GSI of adult male fish [67]–[69]. The reproductive toxicity of leachate and treated effluent was certified by the testicular atrophy of males in this study. These significantly lower GSIs are likely to be a result of Vtg synthesis. Jobling et al. [70] reported the inverse correlation between plasma Vtg and GSI in male roaches (*Rutilus rutllus*). Considering that Vtg synthesis is an energy-wasting process, the reproduction and growth of exposed fish tends to be delayed.

The method using estrogen equivalent quantity (EEQ) is recommended for hazard assessment of different classes of estrogenic chemical mixtures. By the use of this approach, Muller et al. [71] assessed the removal efficiency of estrogens by a municipal wastewater treatment plant and found that the biological treatment process possessed the highest removal ratio. The overall estrogenic equivalents of a mixture are defined by the concentration of individual compounds in a mixture times their relative potencies to E_2_, as reported by Safe [72]. Thus, the overall estrogenic potency of a complex mixture, for example, leachate, can be calculated and compared. According to previous studies, the estrogen equivalent factors of the detected EEs were 5.6×10^−4^ for BPA, 6×10^−5^ for OP, 2.4×10^−5^ for D_n_BP, 2.0×10^−7^ for DEHP, and 0.5×10^−7^ for DEP [17], [73]–[76]. The EEQs of leachate, MBR outlet, and treated effluent when calculated by this method were 3.78 ng/L, 0.008 ng/L, and 0.01 ng/L, which seemed to be relatively low. However, remarkable estrogenic activities that led to the induction of Vtg, and depression of *γ*-GTP activity and GSI were observed in leachate and treated effluent exposed male fish. The biological responses in male goldfish did not agree either with the chemical data obtained by GC-MS. Similar results were also reported by Blankvoort et al. [60], and Gunnarsson et al. [77]. Leachate is supposed to compromise the validity of the EEQ approach based on chemical analysis because of the cross talk between multiple endocrine pathways [72], and hence it should be noted that an underestimation may be made if only using the results of chemical analysis. However, *in vivo* bioassays mostly provide an integrated result of the total potency of complex mixtures to induce some biological responses, just as mentioned above that due to the presence of some substances with androgenic activities, the plasma T levels were unexpectedly not changed. Thus batteries of biomarkers at the molecular level, biochemical level, and tissue level, may prove more useful than single biomarkers in characterizing certain endocrine disrupting effect. Accordingly such *in vivo* experimentation might be complicated to operate and costly in financial terms, thereby making it valuable in study rather than in routine controls. Taking these findings together, a combination of chemical analysis and an *in vivo* bioassay is necessary to provide comprehensive knowledge and valid assessments of estrogenicity of a complicated mixture, such as landfill leachate.

Based on the comprehensive assessment results, the efficiency of unit operations in removing the previously selected compounds can be well evaluated. Due to the heavily contaminated property and poor biodegradability of landfill leachate, conventional wastewater treatment cannot fulfill treatment requests. Advanced wastewater treatments, especially the membrane process, for example, reverse osmosis treatment, are widely applied as a landfill leachate treatment. Coors et al. [10] used a reporter-gene-based bioassay with a human breast-cancer-derived cell line to study the removal rate of two different membrane employing processes and found that the concentrated effluent of the reverse osmosis treatment still showed an estrogenic response. The selected leachate treatment facility employed a “membrane bioreactor+reverse osmosis+aeration zeolite biofilter” treatment process, with qualified removal ratios of conventional parameters (i.e., COD_Cr_, BOD_5_, TN, TP, and TSS), and the quality of the treated effluent meets Grade one A standard of the *Discharge Standard of Pollutants for Municipal Wastewater Treatment Plant* (GB18918-2002, China). A relatively high rejection ratio of detected EEs, which can reach 87.2% or above, was achieved by the landfill leachate treatment process. However, the treated effluent still showed estrogenic activity in bioassays, indicating the existence of environmental estrogens and the potential risk to the aquatic habitat.

## Conclusions

To achieve both qualitative and quantitative analysis results for estrogenic potency of leachate and treated effluent, a combined approach incorporating two types of analyses was proposed and applied to a real case study in Qingdao. The following are the main findings: the method combined chemical techniques with the responses of test organisms can be effectively applied for analyzing estrogen compounds, and resulted in a more comprehensive characterization of the estrogenic activity of environmentally complex samples. In a concentration of 200 times diluted, the leachate still showed strong estrogenic activity and led to bio-toxicity effects on male goldfish. The removal rate of “membrane bioreactor+reverse osmosis+aeration zeolite biofilter” treatment process for the target EEs can reach 87.2% or above; however, 5 types of EEs were still present in the effluent and contribute to an estrogen-like effect.

## Supporting Information

Table S1 Biological responses of goldfish exposed to different dilutions of leachate. Male goldfish (12 fish each group) were exposed to different dilutions of the leachate (1∶10, 1∶20, 1∶50, 1∶100, and 1∶200) in a semi-static exposure system after acclimated in the lab for 14 days. The leachate was diluted with dechlorinated tap water and a negative control group (dechlorinated tap water) was also set up. The mortality rate, survival time, and abnormal behavior were recorded.
(DOCX)

Table S2 The ARRIVE guidelines checklist for animal research: reporting *in vivo* experiments.
(PDF)
