# Supplementary material for: An Integrated Approach Combining Chemical Analysis and an In Vivo Bioassay to Assess the Estrogenic Potency of a Municipal Solid Waste Landfill Leachate in Qingdao
Source: PLoS One. 2014 Apr 17;9(4):e95597. doi: 10.1371/journal.pone.0095597 (PMC3990707; doi:10.1371/journal.pone.0095597)
Supplement: Table S1 — Biological responses of goldfish exposed to different dilutions of leachate. Male goldfish (12 fish each group) were exposed to different dilutions of the leachate (1∶10, 1∶20, 1∶50, 1∶100, and 1∶200) in a semi-static exposure system after acclimated in the lab for 14 days. The leachate was diluted with dechlorinated tap water and a negative control group (dechlorinated tap water) was also set up. The mortality rate, survival time, and abnormal behavior were recorded. (DOCX) [file pone.0095597.s001.docx]

**Table S1.** **Biological responses of goldfish exposed to different dilutions of leachate**

| Dilutions of the leachate | Mortality rate | Survival time | abnormal behavior |
| --- | --- | --- | --- |
| 1:10 | 100 % | 10 min | Yes |
| 1:20 | 100 % | 1 hour | Yes |
| 1:50 | 100 % | 4 hours | Slightly |
| 1:100 | 100 % | 14 hours | Slightly |
| 1:200 | 0 | More than 28 days | none |
